# Xanthogranuloma of the Eyelid: A Case Report

**DOI:** 10.7759/cureus.42477

**Published:** 2023-07-26

**Authors:** Estefania Ramirez Marquez, Sofía C Ayala Rodríguez, Edgar De Jesús Rodríguez, Jose Raul Montes, Miguel A Noy Malave, Armando L Oliver

**Affiliations:** 1 Ophthalmology, University of Puerto Rico School of Medicine, Medical Sciences Campus, San Juan, USA; 2 Pathology, Laboratorio Patología Dr. Noy, San Juan, USA

**Keywords:** surgical intervention, nasal polyps, orbital mass, yellow plaques, necrobiotic xanthogranuloma

## Abstract

We report on the case of a Hispanic woman with necrobiotic xanthogranuloma (NBX) whose disease was managed based on her symptoms. She underwent a diagnostic and debulking surgical intervention and surveillance for hematologic malignancy. This 56-year-old patient presented with a six-year history of enlarging masses and swelling around her eyes, with intermittent inflammation, associated pain, and occasional redness. Her past medical history was remarkable for asthma and nasal polyps. Upon external examination, she had severe fullness of the upper lids with yellow plaques and palpable masses along them, nontender palpation, the absence of visible erythema, and blepharoptosis in both eyes. The patient presented with bilateral visual field constriction due to mechanical obstruction. An orbital computed tomography scan revealed a dense diffuse lesion involving the pre- and postseptal tissues and invading the orbit of the right eye. A facial magnetic resonance imaging scan revealed infiltration of the postseptal spaces within both orbits. A skin and soft tissue biopsy from the bilateral periorbital regions of both eyes confirmed the diagnosis of NBX. A workup for underlying hematologic malignancies, including plasma cell dyscrasias and lymphoproliferative disorders, was unremarkable. The patient underwent diagnostic and debulking surgery in an attempt to improve her visual function. Subsequently, she was scheduled for ongoing monitoring of her disease progression.

## Introduction

Necrobiotic xanthogranuloma (NBX) is characterized by yellow plaques and a slowly disfiguring process with a predilection for the periorbital tissues [1-5]. This rare disease has an unknown etiology, although a strong association with paraproteinemia has been established [1-5]. A good number of patients with NBX have previously been diagnosed with monoclonal gammopathy of unknown significance, multiple myeloma, lymphoma, or leukemia [1,2,5].

Treatment options for managing NBX are determined based on whether the course of the patient’s disease is associated with an underlying malignancy [2]. Therapies reported in the literature include corticosteroids, interferon alpha, infliximab, intravenous immunoglobulin, alkylating agents, laser, radiotherapy, and surgery [1-5]. The literature about NBX is limited and heterogeneous; however, a timely diagnosis and monitoring are essential if patients are to be provided with the best care possible, as this condition may serve as a warning of a life-threatening malignancy [2]. We hereby present the case of a Hispanic woman with NBX whose disease was managed with diagnostic and debulking surgery as well as surveillance for malignancy.

## Case presentation

**Figure 1:**
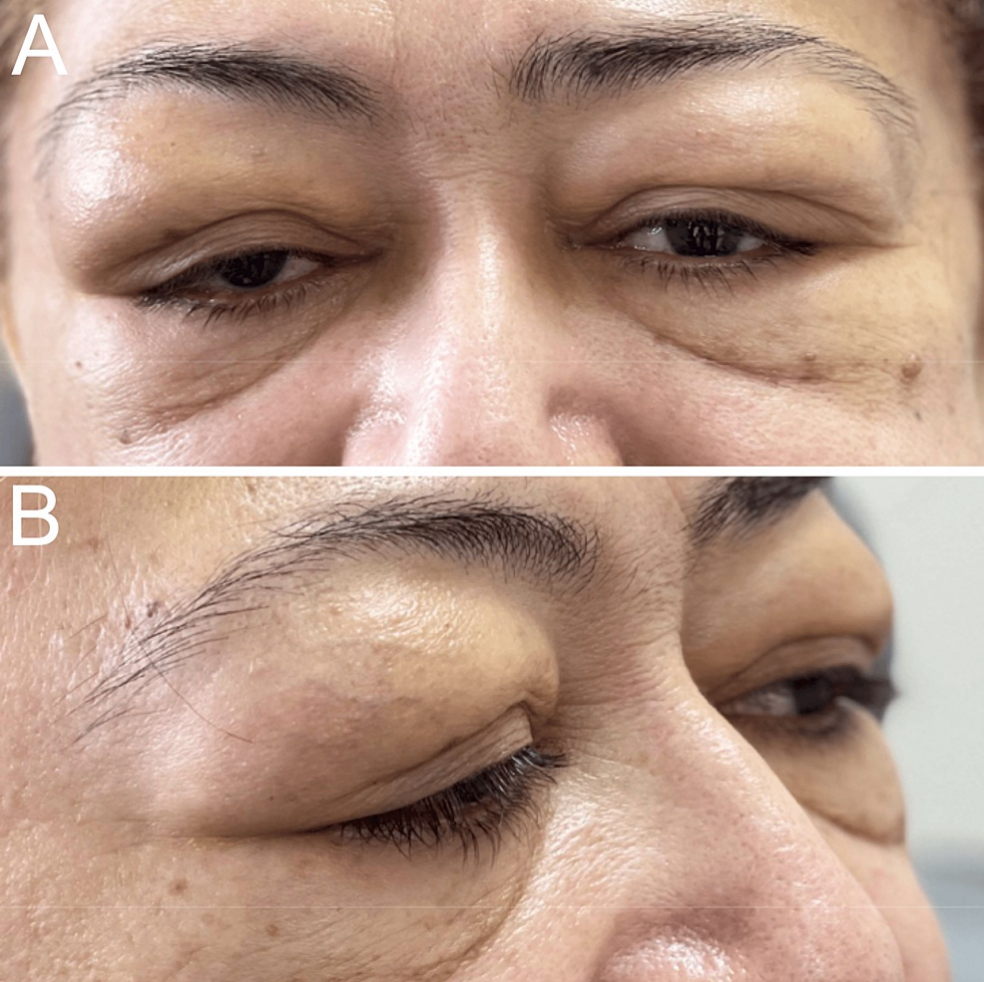
(A) The anterior view and (B) lateral view show fullness in both eyes and upper eyelids, accompanied by yellow pigmentation.

A 56-year-old Hispanic female presented with a six-year history of enlarging masses and swelling around both eyes (oculus uterque or OU), with intermittent inflammation, associated pain, and occasional redness (Figure 1). Her past medical history was remarkable for hypertension and asthma with associated nasal polyps. Her systemic conditions were controlled with amlodipine and albuterol. The patient delayed seeking treatment due to problems with medical insurance, transportation, and the COVID-19 global pandemic. Her review of systems and her past social and family histories were otherwise unremarkable.

**Figure 2:**
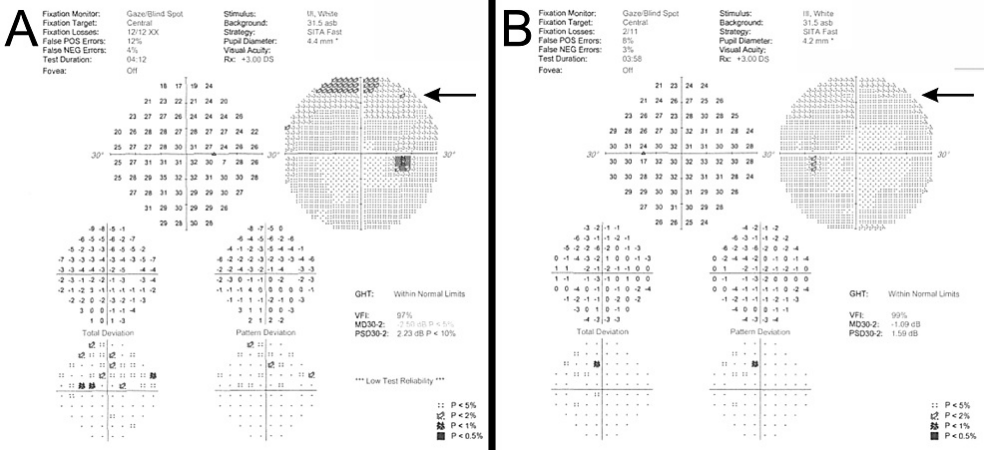
Bilateral visual field upon presentation (Humphrey, Central 30-2 threshold test, stimulus III, white, Swedish Interactive Threshold Algorithm-Fast). (A) Right and (B) left visual fields reveal superior field defect (arrows) due to bilateral mechanical ptosis.

Upon a comprehensive ophthalmic evaluation, her best-corrected visual acuity was 20/25 in the right eye (oculus dexter or OD) and 20/20 in the left eye (oculus sinister or OS). The intraocular pressure was 16 mmHg OU. The pupils were round and reactive to light, and there was no afferent pupillary defect. Color vision, as assessed by the Ishihara color plate test, revealed no defect OU. Extraocular movements were within normal limits. The margin to reflex distances 1 (MRD1s), measured from the light reflex on the patient’s cornea to the level of the center of the upper eyelid margin with the patient gazing straight ahead, were 0.5 mm OD and 1.0 mm OS. The MRD2s, measured from the light reflex on the patient’s cornea to the level of the center of the lower eyelid margin with the patient gazing straight ahead, were 5.0 mm OD and 5.0 mm OS. The levator functions were 10 mm OD and 13 mm OS. Her Hertel exophthalmometric values (base 98) were 21.5 OD and 21.0 OS. Upon external examination, she had severe fullness of the upper lids, palpable masses and yellow plaques along the upper lids, nontender palpation, no visible erythema, and blepharoptosis OU. A slit-lamp examination was within normal limits, bilaterally, with no evidence of keratic precipitates, signs of inflammation in anterior chambers, or vitreous cells in either eye. The patient's fundus examination was unremarkable in both eyes (OU). A Humphrey visual field analysis revealed a superior field defect due to mechanical ptosis OU (Figure 2).

**Figure 3:**
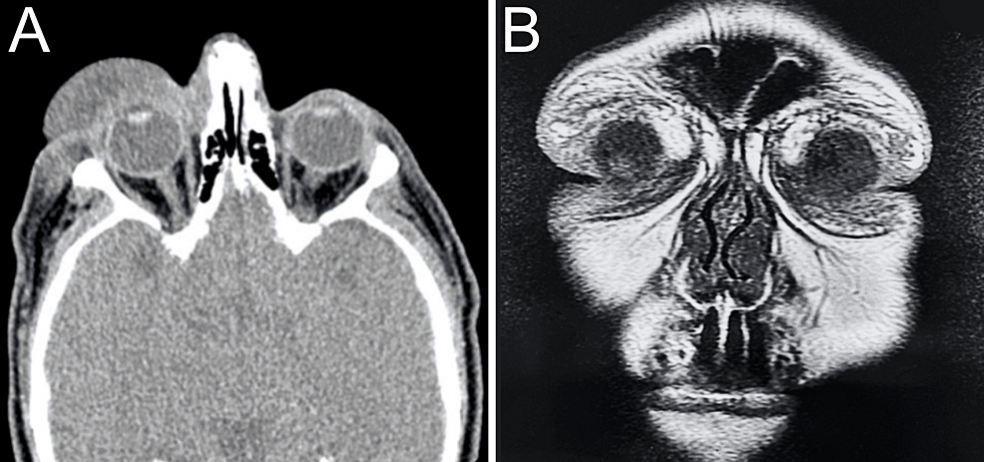
Orbital computed tomography and magnetic resonance imaging of the patient. (A) Orbital computed tomography reveals that the preseptal space was completely infiltrated by the lesion. (B) Magnetic resonance imaging confirms the infiltration of the postseptal spaces within both orbits.

**Figure 4:**
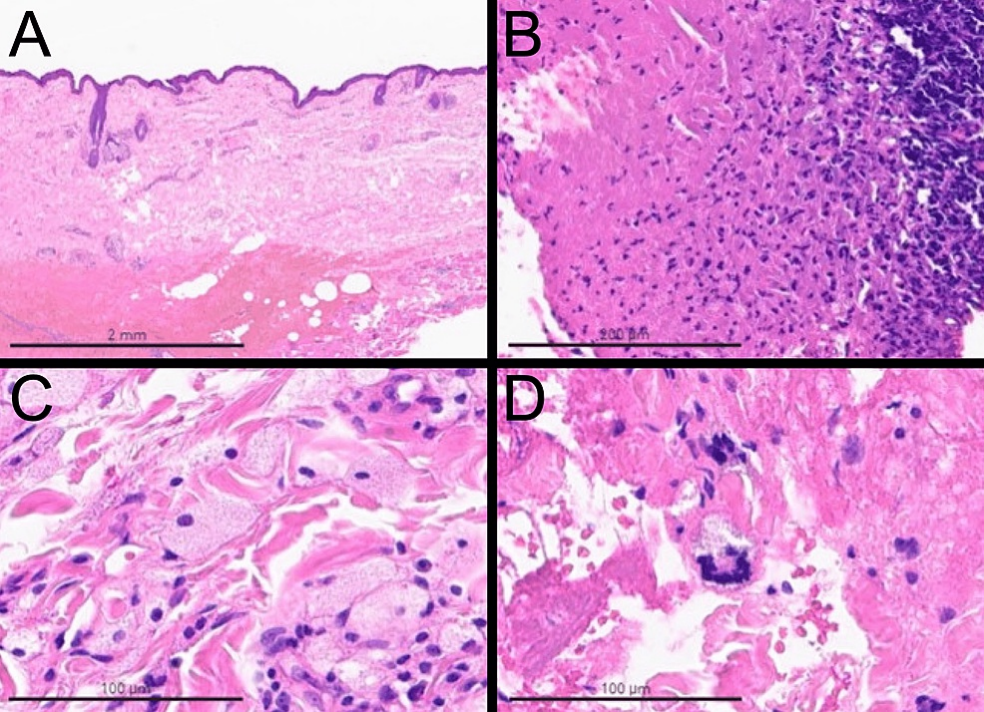
Histopathology of the periorbital mass. (A) Normal epidermis and underlying broad zones of altered collagen (necrobiosis) (H&E stain, 1×). (B) Altered collagen (necrobiosis) surrounded by a mixed inflammatory cellular infiltrate (H&E stain, 20×). (C and D) Numerous foamy macrophages and giant cells are at the border of a zone of altered collagen (H&E stain, 40×). H&E, hematoxylin and eosin

An orbital computed tomography scan revealed a dense diffuse lesion involving the pre- and postseptal tissues and invading the orbit OD (Figure 3A). A facial magnetic resonance imaging scan revealed infiltration of the postseptal space within both orbits (Figure 3B). A skin and soft tissue biopsy from the bilateral periorbital region OU provided results consistent with NBX (Figure 4). A workup was done by a hematology-oncology specialist to rule out secondary etiologies, such as plasma cell dyscrasias or lymphoproliferative disorders. Studies, including a complete blood cell count (CBC), a comprehensive metabolic panel (CMP), the immunotyping and protein electrophoresis of serum, urine protein electrophoresis, and immunoglobulin A (IgA), IgG, and IgM tests, were unremarkable. Beta-2 microglobulin was measured at 2.79 mg/dL, which exceeded the expected range for the general population. Free kappa level and kappa to lambda ratio were found to be 22.51 and 1.748 mg/dL, respectively, indicating an elevation beyond the normal range.

The patient underwent a comprehensive diagnostic and debulking surgery; however, this procedure was aborted to prevent provoking damage, as the abnormal tissue had infiltrated the normal tissue. She was later scheduled for continued monitoring of her disease progression as well as for a follow-up with a hematology-oncology specialist for malignancy surveillance.

## Discussion

NBX is a rare disease that presents multiple challenges in clinical practice. This condition belongs to a group of adult xanthogranulomatous disorders, including adult-onset xanthogranuloma, Erdheim-Chester disease, adult-onset asthma, and periocular xanthogranuloma [6]. Limited information is available regarding an association between respiratory diseases and adult xanthogranulomatous disorders; however, it is interesting to note that this patient had a history of asthma and nasal polyps [6]. Histopathologically, NBX is distinguished by the presence of foamy histiocytes, Touton giant cells, and collagen necrosis [1,2]. Patients with NBX may present xanthomas around the eyes and eyelids, ranging from yellow to orange; orbital masses; and ulceration [1-6]. Over time, cutaneous lesions may progress, leading to complications such as proptosis, restrictive strabismus in the orbit, ptosis, ectropion, and lagophthalmos of the periorbital region [2].

The management of NBX involves a variety of clinical strategies, with the selection of the specific treatment to be used depending on the patient’s unique clinical presentation, disease extent, and underlying disease [2,6]. The diagnosis of NBX should prompt the evaluation of hematologic malignancies, such as multiple myeloma, monoclonal gammopathy of undetermined significance, Waldenström macroglobulinemia, and chronic lymphocytic leukemia, as there exists a known association between these diseases [1-6]. In the case of the patient described in this research, studies to rule out hematologic diseases, including a CBC, a CMP, immunotyping, protein electrophoresis of serum, urine protein electrophoresis, and IgA, IgG, and IgM tests, were found to be unremarkable. Beta-2 microglobulin, free kappa, and the kappa-to-lambda ratio were slightly elevated compared to the normal range for the general population. Although these findings are not suggestive of an active plasma or lymphoproliferative disorder, the abnormal light chain values may warrant further surveillance. Elevated beta-2 microglobulin may suggest the presence of hematologic disease, kidney disease, infection, or an autoimmune or inflammatory disorder; therefore, this parameter is not specific enough to identify any single medical condition [7]. This patient did not undergo a bone marrow biopsy; however, it would seem a useful tool to include in future surveillance of hematologic disease. 

Alternatively, surgical intervention may be considered when the lesions progress and cause significant complications, which could include any or all of the following: ptosis, lagophthalmos, and decreased vision. The patient described in this case had significant visual field constriction (Figure 2). Diagnostic and surgical debulking of the orbital lesions was carried out on this patient to improve her visual function. In the absence of complications, such as those described, surgical debulking might better be discouraged as there have been high rates of recurrence reported with this technique.

NBX is a progressive disease that increases the patient's risk of developing hematologic malignancies. Patients with NBX require lifelong close monitoring [1,2]. There is a lack of certainty regarding the specific studies that need to be repeated and the optimal timing for follow-up. Nevertheless, physicians should tailor management strategies to patients’ findings. It is also important to offer patients with NBX reproductive counseling as the disease is inherited in an autosomal recessive pattern [8]. Given the unpredictable nature of the disease course, patients must receive education and guidance to work toward positive outcomes. Further studies should be conducted to develop improved algorithms for monitoring malignancies and preventing adverse ocular outcomes in patients with NBX. Additionally, the associations between respiratory diseases such as asthma and nasal polyps and NBX should also be further explored.

## Conclusions

NBX is a progressive disease that presents with xanthomas around the eyes and eyelids, ranging from yellow to orange; orbital masses; and ulceration. The case suggests that strategies managing this condition involve a personalized approach that considers individual underlying diseases and symptoms. The diagnosis of NBX should prompt an evaluation for hematologic malignancies; the condition itself requires lifelong monitoring. The associations between NBX and such respiratory conditions as asthma and nasal polyps should be further explored.
